# Prolonged phloem ingestion by *Diaphorina citri* nymphs compared to adults is correlated with increased acquisition of citrus greening pathogen

**DOI:** 10.1038/s41598-018-28442-6

**Published:** 2018-07-09

**Authors:** Justin George, El-Desouky Ammar, David G. Hall, Robert G. Shatters, Stephen L. Lapointe

**Affiliations:** 1 USDA-ARS, Subtropical Insects and Horticultural Research Unit, United States Horticultural Research Laboratory, Fort Pierce, Florida USA; 2 University of Florida, IFAS, Lake Alfred, Florida USA

## Abstract

Citrus greening disease (huanglongbing), currently the most destructive citrus disease worldwide, is putatively caused by *Candidatus* Liberibacter asiaticus (CLas), a phloem-limited bacterium transmitted by the Asian citrus psyllid *Diaphorina citri*. Electrical penetration graph (EPG) recordings over 42 h were performed to compare the feeding behavior of *D. citri* adults and 4^th^ or 5^th^ instar nymphs feeding on CLas-infected or healthy citron plants. Nymphs performed more individual bouts of phloem ingestion (E2) and recorded longer phloem ingestion total time compared with adults, whereas adults performed more bouts of xylem ingestion (G) and recorded greater total time of xylem ingestion compared with nymphs. Quantitative polymerase chain reaction tests indicated that 58% of nymphs and 6% of adults acquired CLas during the 42 h EPG-recorded feeding on infected plants. In a histological study, a greater proportion of salivary sheaths produced by nymphs were branched compared to those of the adults. Our results strongly suggest that more bouts and longer feeding time in the phloem by nymphs may explain their more efficient CLas acquisition from infected plants compared to adults. This is the first EPG study comparing nymphs and adults of *D. citri* on healthy and infected citrus plants in relation to CLas acquisition.

## Introduction

The Asian citrus psyllid, *Diaphorina citri* Kuwayama (Hemiptera: Liviidae), is the primary vector of the phloem-limited bacterium *Candidatus* Liberibacter asiaticus (CLas), the putative causal agent of citrus greening disease, also known as huanglongbing (HLB). HLB is currently the most serious and destructive citrus disease in the USA, Brazil, Central America and most other citrus growing areas in the world^1–3^. CLas can infect all commercial citrus cultivars, resulting in major reductions in fruit quality, yield^4^ and lifespan of infected trees^5^. HLB has spread quickly in Florida-USA since its first detection in 2005, and is now threatening citrus production in Texas, California and other parts of the USA^6^. The economic effects of the spread of HLB throughout the state of Florida have been devastating: as a result of the disease, citrus production dropped from 242 million boxes in 2005 to 68.8 million boxes in 2014^7^.

CLas is acquired in a persistent, circulative-propagative manner by both *D. citri* nymphs and adults, but nymphs are much more efficient than adults in the acquisition of CLas from infected citrus plants^8–11^. In two studies, from Japan and Florida-USA, in which *D. citri* nymphs and adults were compared^9,11^, the rate of CLas acquisition by nymphs ranged between 49–78%, whereas acquisition by adults ranged between 8–29% following acquisition access periods (AAPs) of 1–7 days for nymphs and 1–21 days for adults on infected citrus. Furthermore, in these two studies, only CLas acquired by *D. citri* during the nymphal stage was successfully transmitted to healthy citrus plants. In another study in Florida, 60–100% of *D. citri* acquired CLas during the nymphal stage, whereas only 39% acquired it during the adult stage after 35 days of AAP on infected plants^10^. Possible reasons for the greater efficiency of *D. citri* nymphs to acquire and transmit CLas from infected citrus, compared to adults, are unknown, but differences in the innate immunity between nymphs and adults have been suggested based on a proteomic study of healthy and CLas-infected psyllids^12^. The great differences between nymphs and adults in the acquisition and transmission of CLas have important implications for the epidemiology and control of this serious citrus disease^2,3,6^.

Previous studies using the electrical penetration graph (EPG) technique indicated that CLas can only be acquired by *D. citri* after ingestion from the phloem of infected plants^13,14^. Bonani *et al*.^13^ showed that this bacterium was not acquired, neither during stylet pathway through the epidermis and parenchyma (waveform C), nor during waveforms D (first contact with the phloem) or E1 (phloem salivation). In contrast, 6% of psyllid adults tested positive for CLas by qPCR when allowed to perform an E2 waveform for a period of 1 h, showing that bacterial acquisition occurred only during phloem ingestion (E2). Luo *et al*.^14^, also using EPG, showed that *D. citri* adults can acquire CLas after E2 waveforms as short as 2 min. However, to our knowledge, no EPG studies have been performed so far on *D. citri* nymphs or to examine if differences in feeding behavior between nymphs and adults can account for the greater ability of *D. citri* nymphs to acquire CLas from infected plants compared to adults.

In the present work, we recorded the feeding behavior of both nymphs and adults of *D. citri* on healthy and CLas-infected citrus plants using EPG for an extended period (42 h). We then tested all the psyllids by qPCR to correlate their feeding behavior with CLas acquisition. Highly significant differences in the frequency and duration of the phloem ingestion phase were found between psyllid nymphs and adults that may account for the greater ability of nymphs to acquire CLas from infected citrus.

## Results

### Electrical Penetration Graph Recordings of *D. citri* nymphs and adults

**Figure 1:**
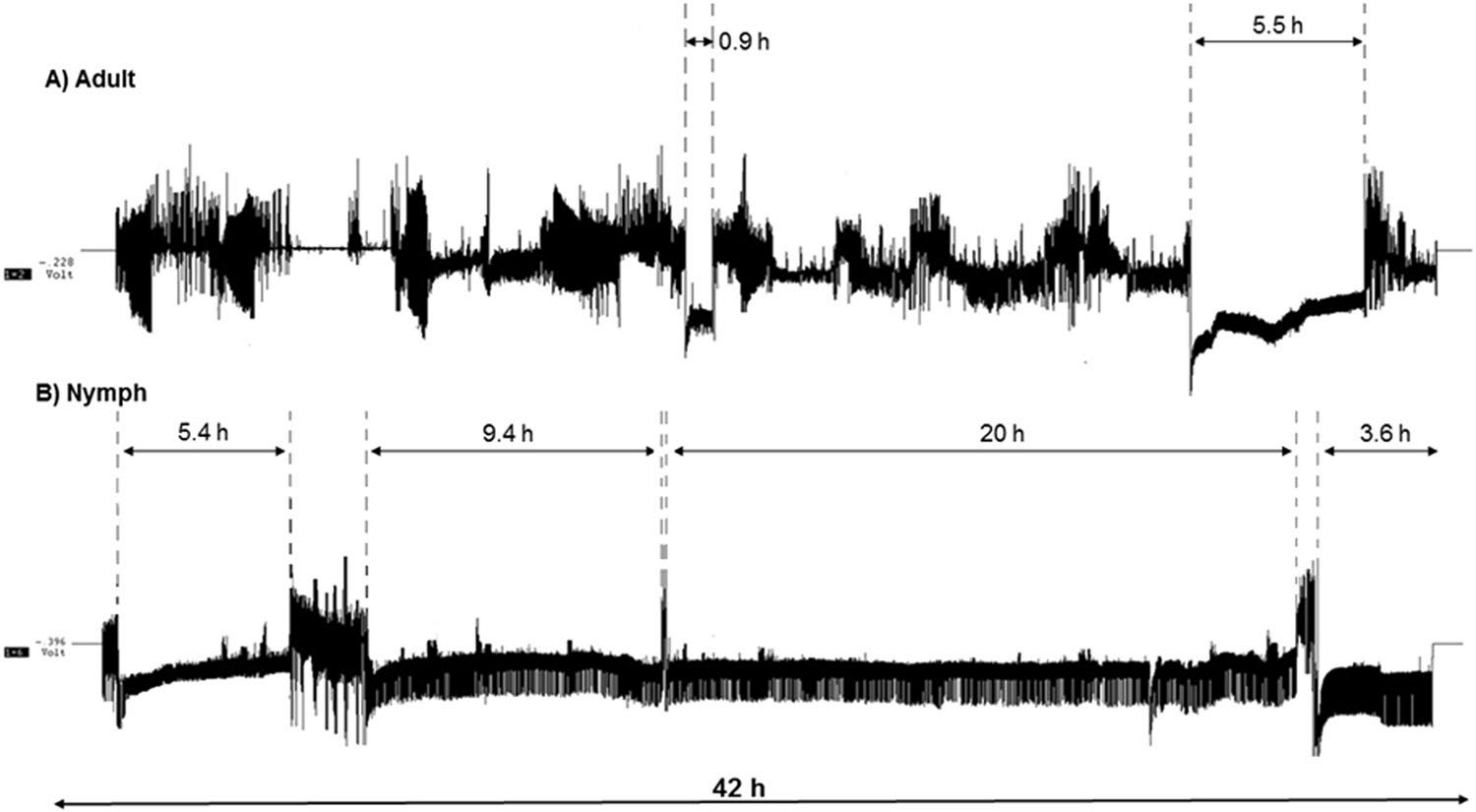
Examples of electrical penetration graph recordings showing feeding activities of *Diaphorina citri* adults (**A**) and nymphs (**B**) during 42 h recordings. Vertical dashed lines with double arrows indicate periods of continuous phloem ingestion (waveform E2).

Waveforms produced by *D. citri* nymphs and adults during the 42 h recording periods were generally similar to those reported previously for *D. citri* adults^13,15^. These waveforms were readily classified, according to the classification of Bonani *et al*.^13^. Overall, *D. citri* nymphs spend significantly greater time in phloem ingestion activities compared to the adults (Fig. 1).

#### Frequency of phloem feeding activities is higher in nymphs vs. adults

**Figure 2:**
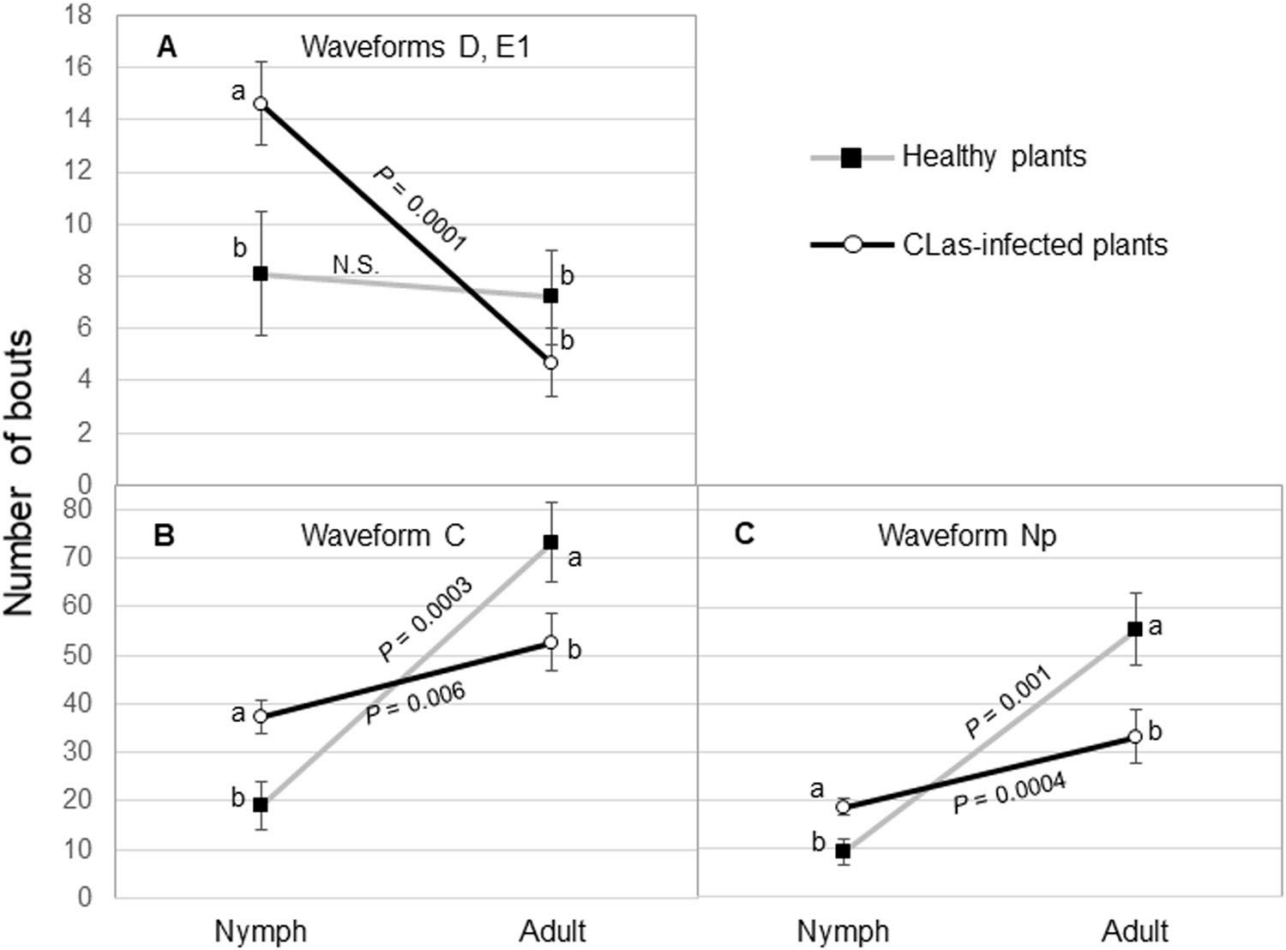
Interaction plots of mean number of bouts (±SEM) of feeding activities during 42 h recordings *by Diaphorina citri* nymphs and adults on young leaves of healthy (*n* = 18) or CLas-infected (*n* = 33) citron plants. Means within insect stage with different letters are significantly different (ANOVA, α = 0.05). *P* values indicate the comparison (t test) of nymphs and adults within plant infection status for each waveform.

Significant interactions were observed between psyllid stage (nymph vs. adult) and plant infection status (healthy vs infected) on the number of bouts (frequency) of waveforms corresponding to intercellular passage (C, aka stylet pathway), phloem penetration (D), phloem salivation (E1) and non-probing (Np) activities (Table 1). There were significant interactions between psyllid stage and infection status for C, D, E1 and Np waveforms (*P* < 0.01). The number of bouts of D and E1 were often same, because phloem salivation follows immediately upon phloem penetration. As a result, one interaction plot is presented (Fig. 2A) for waveforms D and E1. The infection status of the plant had no effect on the number of D or E1 bouts conducted by adult *D. citri*. However, the number of bouts of D and E1 was significantly greater for *D. citri* nymphs feeding on CLas-infected plants compared with nymphs feeding on healthy plants (Fig. 2A). On infected plants, nymphs conducted more D and E1 probes compared with adults, while the number of D and E1 bouts on healthy plants was equivalent for nymphs and adults. More bouts of waveform C (intercellular passage) were recorded by nymphs on CLas-infected plants compared with nymphs on healthy plants (Fig. 2B). Adult psyllids showed an inverse response as more bouts of C were recorded by adults on healthy plants compared with adults on CLas-infected plants. Significantly fewer C bouts were recorded by nymphs compared with adults on healthy plants. Periods of non-probing (Np) recorded by nymphs and adults showed a similar pattern as that for waveform C. Adults recorded a greater number of non-probing bouts on healthy plants compared with adults on infected plants while nymphs recorded more Np bouts on infected plants compared with nymphs on healthy plants. There was no significant interaction between psyllid stage and plant infection status for the number of bouts of E2 (phloem ingestion, *P* = 0.11) or G (xylem ingestion, *P* = 0.52). There was no effect of plant infection status on the number of E2 bouts (*P* = 0.86). There was an effect of insect stage on the number of E2 bouts (*P* = 0.006) with fewer mean (±SEM) number of bouts of phloem ingestion performed by adults (5 ± 1 bouts) compared with nymphs (9 ± 1 bouts). There was a significant effect of plant infection status on the number of bouts of xylem ingestion (*P* = 0.02) and a larger effect of insect stage (*P* = 0.0001) (Table 1). More bouts of G waveform were recorded from CLas-infected plants (10 ± 1 bouts) compared with healthy plants (6 ± 1 bouts). Adults engaged in more bouts of xylem ingestion (14 ± 1 bouts) compared with nymphs (3 ± 1 bouts) (Table 1). Nymphs of *D. citri* engaged in more bouts of phloem ingestion and fewer bouts of xylem ingestion compared with adults.

**Table 1:** Effect of psyllid stage and CLas infection on mean (±SEM, *n* = 33 for infected, 18 for healthy) frequency (no. of bouts) of feeding activities by *Diaphorina citri* adults and nymphs on citron during 42 h EPG recordings.

Waveform*	Effect	Psyllid stage		Plant infection		*F*-value	*P* > *F*
		Nymph	Adult	Healthy	Infected		
C	Stage	32 ± 4	60 ± 4			33.1	**0.001**
	Plant infection			49 ± 5	45 ± 3	0.03	0.84
	Interaction					10.1	**0.002**
D	Stage	13 ± 1	6 ± 1			8.7	**0.004**
	Plant infection			8 ± 1	10 ± 1	1.2	0.27
	Interaction					6.1	**0.01**
E1	Stage	13 ± 1	6 ± 1			8.7	**0.004**
	Plant infection			8 ± 1	10 ± 1	1.2	0.27
	Interaction					6.1	**0.01**
E2	Stage	9 ± 1	5 ± 1			7.7	**0.006**
	Plant infection			6 ± 1	7 ± 1	0.02	0.86
	Interaction					2.6	0.11
G	Stage	3 ± 1	14 ± 1			39.7	**0.0001**
	Plant infection			6 ± 1	10 ± 1	5.4	**0.02**
	Interaction					0.4	0.52
Np	Stage	16 ± 4	41 ± 4			33.4	**0.0001**
	Plant infection			35 ± 4	26 ± 3	1.5	0.22
	Interaction					9.1	**0.003**

#### Duration of phloem feeding activities is much longer in nymphs vs. adults

**Figure 3:**
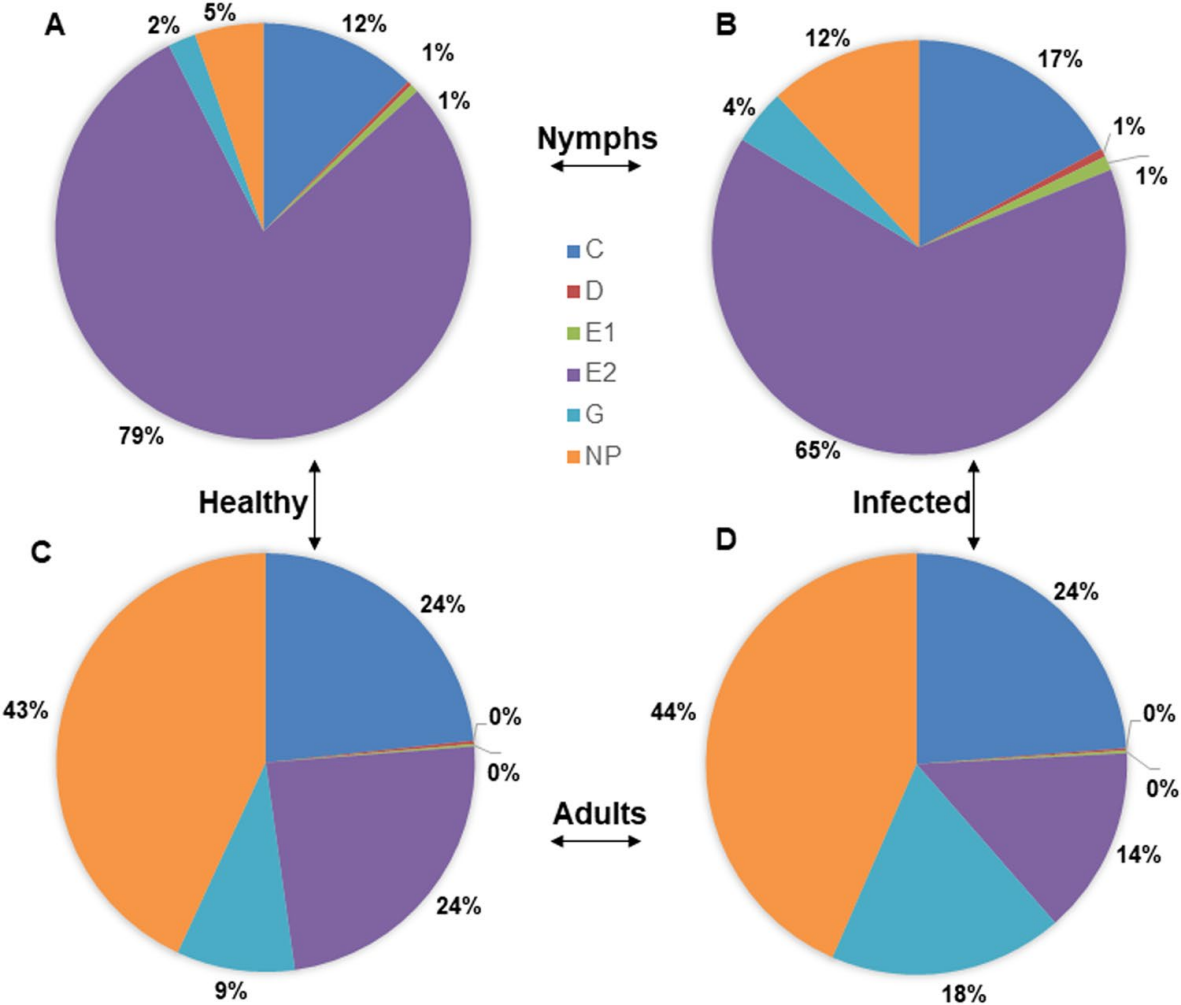
Percent duration of each feeding activity performed by *Diaphorina citri* nymphs (**A** and **B**) and adults (**C** and **D**) feeding on young leaves of healthy (**A** and **C**) or CLas-infected (**B** and **D**) citron plants during 42 h recordings. Phloem ingestion (E2) was longer in nymphs compared with adults whereas druation of xylem ingestion (G) was greater in adults compared with nymphs.

There were no significant interactions between plant infection status and insect stage, and no effect of plant infection status for the duration of the waveforms recorded (Table 2). Insect stage, however, had a significant effect on the mean duration of all waveforms (Table 2 and Fig. 3). The duration of individual bouts of waveforms C, D, E1 and E2 were significantly longer for nymphs compared with bouts conducted by adults (Table 2). The mean duration (±SEM) of bouts of phloem ingestion (E2) by nymphs (335 ± 41 min) was 4 times greater than that of adults (85 ± 39 min) (*P* = 0.0001) (Figs 1, 3 and Table 2). However, the mean duration (±SEM) of xylem ingestion bouts (G) was significantly longer (*P* = 0.04) for adults (29 ± 3 min) compared with the duration of xylem feeding bouts by nymphs (18 ± 3 min). Also, adults (41 ± 6 min) spent significantly more time in non-probing (Np) activities compared with nymphs (22 ± 6 min) (*P* = 0.02, Table 2).

**Table 2:** Effect of psyllid stage and CLas infection on mean (±SEM, *n* = 33 for infected, 18 for healthy) duration (min) of individual bouts of feeding activities by *Diaphorina citri* adults and nymphs on citron during 42 h EPG recordings.

Waveform*	Effect	Psyllid stage		Plant infection		F-value	Pr > F
		Nymph	Adult	Healthy	Infected		
C	Stage	14.4 ± 1	11.0 ± 1.0			6.6	**0.01**
	Plant infection			12.2 ± 1.3	12.9 ± 0.9	0.08	0.78
	Interaction					1.6	0.22
D	Stage	1.1 ± 0.1	0.6 ± 0.1			19.4	**0.001**
	Plant infection			0.8 ± 0.1	0.8 ± 0.1	0.005	0.94
	Interaction					0.6	0.45
E1	Stage	2.3 ± 0.35	0.6 ± 0.3			10.7	**0.001**
	Plant infection			1.3 ± 0.2	1.5 ± 0.2	0.18	0.67
	Interaction					0.25	0.62
E2	Stage	335.0 ± 41.1	85.2 ± 38.7			20.3	**0.0001**
	Plant infection			220.9 ± 46.1	199.0 ± 32.5	0.36	0.55
	Interaction					0.02	0.88
G	Stage	18.2 ± 3.3	29.2 ± 3.1			4.2	**0.04**
	Plant infection			22.2 ± 3.8	24.8 ± 2.6	0.35	0.55
	Interaction					1.3	0.26
Np	Stage	21.5 ± 5.8	41.1 ± 5.5			5.3	**0.02**
	Plant infection			26.8 ± 6.5	34.0 ± 4.6	0.95	0.33
	Interaction					0.33	0.56

#### Total duration (frequency x mean duration) of phloem and xylem feeding activities

**Figure 4:**
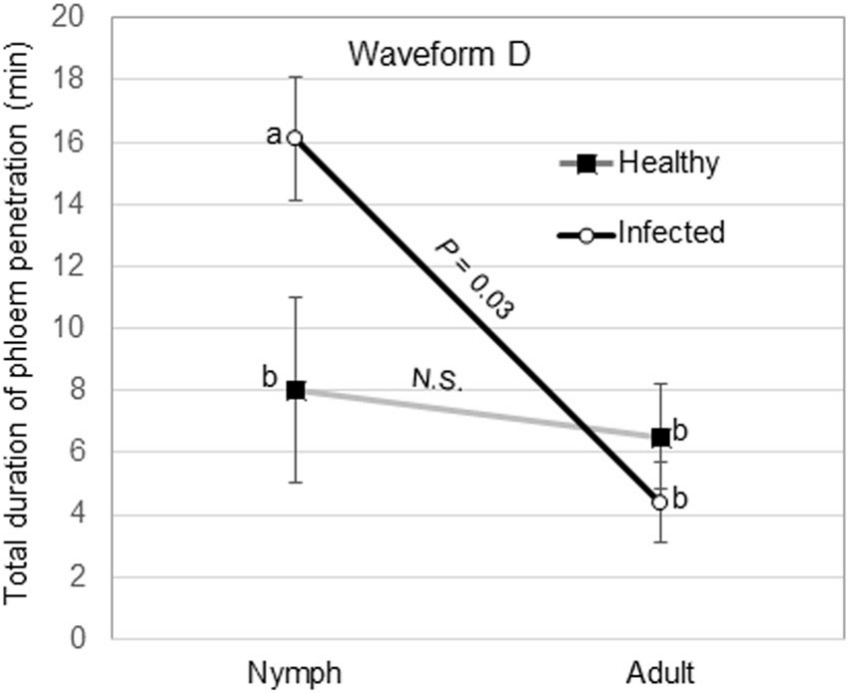
Interaction plot showing the effect of psyllid age and plant infection status on the total duration of phloem penetration activities (D) *by Diaphorina citri* nymphs and adults on young leaves of healthy or CLas-infected citron plants. Means within insect stage with different letters are significantly different (ANOVA, α = 0.05). *P* values indicate the comparison (t test) of nymphs and adults within plant infection status for the waveform.

No significant interaction was observed between psyllid stage and CLas infection status on the total duration of C, E1, E2, G or Np waveforms (Table 3). Plant infection status had no effect on the total duration of intercellular stylet pathway (C), phloem salivation (E1) and non-probing activities (Table 3). However, a significant interaction was observed between the psyllid stage and plant infection status on the total duration of phloem penetration (D); nymphs spent significantly more time in phloem penetration activities on infected plants (16 ± 2 min) compared with nymphs feeding on healthy plants (8 ± 3 min) (*P* = 0.03, Fig. 4).

**Table 3:** Effect of psyllid stage and CLas infection on mean (±SEM, *n* = 33 for infected, 18 for healthy) total duration (frequency × mean duration) of feeding activities by *Diaphorina citri* adults and nymphs on citron during 42 h EPG recordings.

Waveform	Effect	Psyllid stage		Plant infection		F-value	*P* > *F*
		Nymph	Adult	Healthy	Infected		
C	Stage	350 ± 41	593 ± 39			18.1	<**0.001**
	Plant infection			434 ± 47	509 ± 33	1.7	0.19
	Interaction					1.3	0.25
D	Stage	13.6 ± 1.5	5.1 ± 1.4			9.9	**0.002**
	Plant infection			7.2 ± 1.7	10.2 ± 1.2	2.0	0.15
	Interaction					5.8	**0.02**
E1	Stage	23.6 ± 2.4	5.3 ± 2.3			23.5	<**0.001**
	Plant infection			9.5 ± 2.7	16.6 ± 1.9	3.7	0.05
	Interaction					2.8	0.09
E2	Stage	1665 ± 77	448 ± 73			132.0	<**0.001**
	Plant infection			1156 ± 87	979 ± 61	4.8	**0.03**
	Interaction					0.01	0.91
G	Stage	88.7 ± 39.2	373 ± 37			23.9	<**0.001**
	Plant infection			148 ± 44	279 ± 31	6.7	**0.01**
	Interaction					2.3	0.13
Np	Stage	240 ± 66	1090 ± 62			94.5	<**0.001**
	Plant infection			648 ± 74	693 ± 52	0.96	0.33
	Interaction					0.84	0.36

Psyllid stage had a highly significant effect (*P* = 0.01 to 0.001) on the total duration (frequency x mean duration) of all detected waveforms (Table 3). Longer total durations of D, E1 and E2 waveforms were recorded for nymphs compared with adults. Adults spent more time engaged in G, C and Np. Nymphs, on average spent 3.7 times more time engaged in phloem ingestion compared with adults. Adults spent 1.9 times more time engaged in xylem ingestion compared with nymphs (Table 3). Total duration of phloem ingestion by both stages was 1.2 times longer on healthy citron compared to CLas-infected citron (*P* = 0.03, Table 3). On the other hand, the total duration of xylem feeding was 4.2 times longer for *D. citri* adults compared with that of nymphs (Table 3). Also, total duration of xylem feeding was 1.9 times longer on infected plants compared with xylem feeding on healthy plants (Table 3). Overall, the total duration of phloem ingestion was significantly longer on healthy plants; whereas the total duration of xylem feeding was significantly longer on infected plants (Fig. 3A–D). Nymphs spent 79% and 65% of their time in phloem ingestion on healthy and infected plants, respectively (Fig. 3A,B). Adults spent 24% and 14% of their time in phloem ingestion on healthy and infected plants (Fig. 3C,D). The mean duration of phloem ingestion events was significantly longer for nymphs (5.3 h) compared to that of adults (1.3 h) (*P* = 0.0001). Also, it was observed that 100% of nymphs engaged in at least one bout of phloem ingestion during the 42 h recording period, whereas only 72% of adults produced phloem ingestion waveforms. On the other hand, *D. citri* adults performed significantly longer bouts of ingestion from xylem. Adults spent 9 to 18% of the total feeding time in xylem ingestion, whereas nymphs spent only 2 to 4% of their time in xylem feeding activities (Fig. 3A–D). Also, adults spent significantly longer time in non-probing (Np) activities (43–44%) compared to nymphs (5–12%) (Fig. 3A–D).

#### Tested nymphs were much more efficient than adults in acquiring CLas from infected citrus plants

Following DNA extraction from nymphs and adults on which EPG recordings were done, qPCR was performed to test for the presence of CLas. Quantitative PCR of individual psyllids that fed on infected plants showed that 58% (19/33) of nymphs acquired CLas, whereas only 6% (2/33) of adults acquired the CLas pathogen during the acquisition test period (42 h). The mean CT values of CLas associated with nymphs and adults were 30.6 ± 0.8 and 36.5 ± 2.5, respectively, which indicates that CLas titer was significantly higher in infected nymphs than in infected adults (*P* = 0.03). No CLas was detected by qPCR in nymphs or adults that fed on healthy citron plants. Also, no differences were observed in the CT values of CLas within infected leaf samples used in the recordings for adults (32.2 ± 1.3) and nymphs (30.0 ± 1.4) *(P* = 0.94). In the case of CLas-infected (qPCR-positive) nymphs, the shortest phloem ingestion duration (mean) observed was 42 mins, whereas the shortest duration for the two infected adults was 221 min. Fifteen phloem feeding bouts were associated with CLas-infected nymphs, whereas only a single phloem ingestion bout was recorded for infected adults.

### Differences between the salivary sheaths produced by nymphs and adults

**Figure 5:**
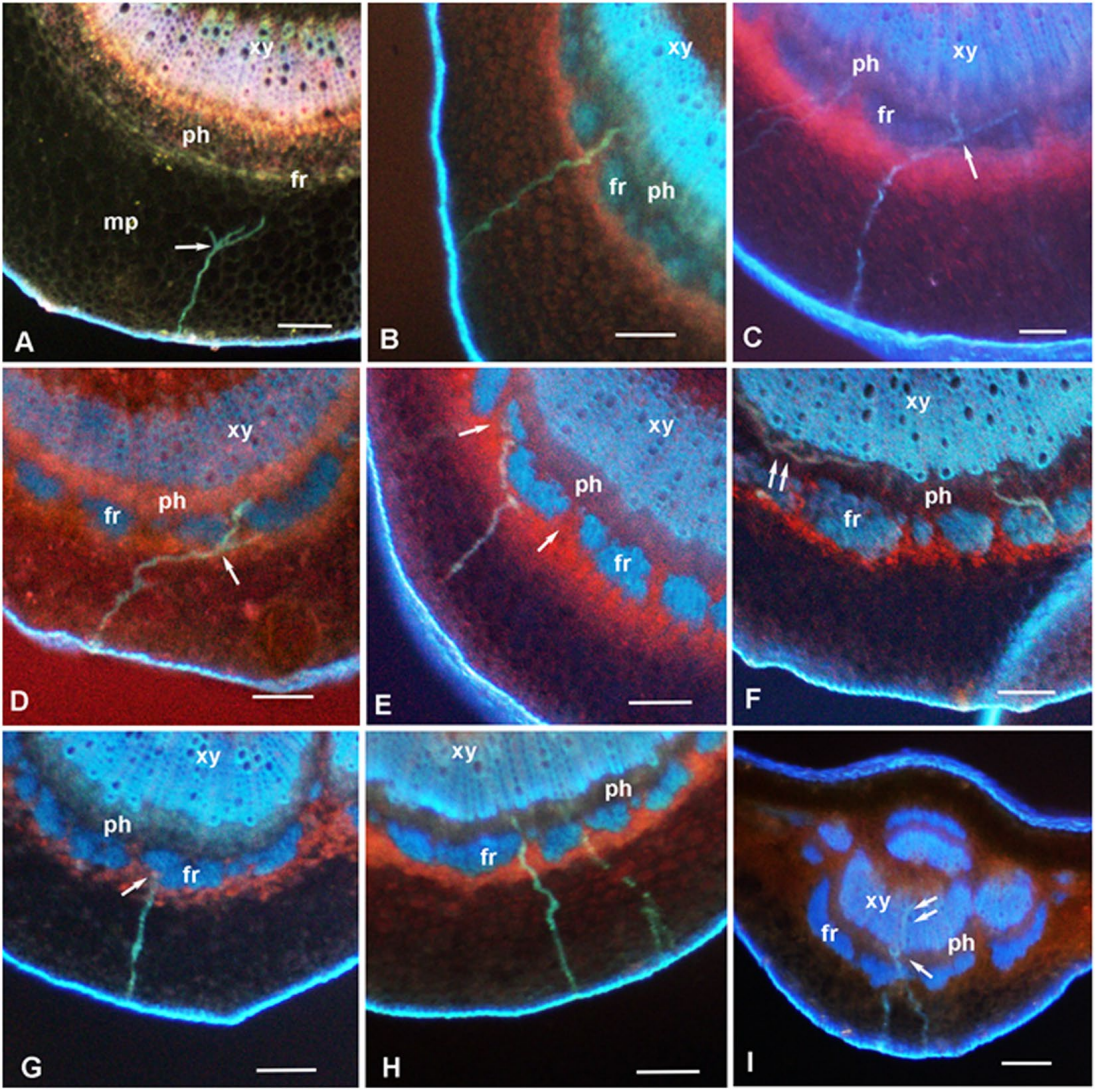
Fluorescence micrographs of stylet sheath tracks (blue-green) produced by *Diaphorina citri* nymphs (**A**–**F**) and adults (**G**–**I**) in midribs of young leaves from healthy or CLas-infected citron plants. Tracks produced by nymphs were often branched (arrows) in mesophyll (**A** and **D**) or in the phloem (**C** and **F**, double arrows). Tracks terminating in phloem are shown in panels B, C, D, F and H; a pathway terminating near the fibrous ring is shown in panel G. In panel I, an adult track included penetration of both phloem (arrow) and xylem (double arrow). In panel E, a track produced by a nymph circumvents the fibrous ring to reach the phloem through gaps (arrows) in that ring. Panels A–C, H and I are from healthy leaves; panels D–F and G are from infected leaves. Colors in panel A are different because this is a younger leaf with the fibrous ring and xylem vessels apparently still immature or unlignified. Abbreviations: fr, fibrous ring; mp, mesophyll parenchyma; ph, phloem; xy, xylem; Scale bars = 100 µm.

The salivary sheaths produced by groups of *D. citri* nymphs or adults feeding on the abaxial side of healthy or CLas-infected citron leaves were examined using fluorescence microscopy of cross sections of the midrib (Fig. 5). Because no differences were found between healthy and infected leaves in sheath branching or in the positions of sheath termini, results of both healthy and infected leaves were pooled (Table 4). No differences were found between nymphs and adults in the proportion of sheaths reaching the vascular bundle, phloem or xylem. However, significant differences were found between nymphs and adults with regard to sheath branching (arrows at Fig. 5A,C and D), where a much higher percentage of nymphal sheaths found in the mesophyll were branched (21.7%) compared to 8.2% of adult sheaths (*P* = 0.046). Also, the proportion of sheaths found at or close to the fibrous ring (arrow at Fig. 5G), believed to form a barrier to stylet penetration of the vascular bundle^15,16^, were much lower with nymphs (18.1%) compared to adults (28%) (*P* = 0.03, Table 4). The percentage of nymphal sheaths that reached the vascular bundle was 43%, compared to 34% by adults (*P* = 0.094).

**Table 4:** Number and proportion (%) of salivary sheaths produced by *Diaphorina citri* nymphs and adults in leaf tissues feeding on midribs of citron leaves. Data pooled for healthy and CLas-infected plants.

Parameter	Nymphs		Adults		X^2^	*P*
	No.	%	No	%		
Branched sheaths/no. sheaths in mesophyll	20/92	21.7	May-61	8.2	3.98	**0.046**
Sheath termini near fibrous ring/total no. sheaths	43/237	18.1	45/161	28	4.8	**0.029**
Sheath termini in VB^1^/total no. sheaths.	102/237	43.1	55/161	34.2	2.8	0.094
Sheaths in phloem/total no. sheaths	92/237	38.8	52/161	32.3	1.49	0.222
Sheaths in xylem/total no. sheaths	10/237	4.2	3/161	1.9	1.02	0.313
Sheaths in phloem/sheaths in vascular bundle	92/102	90.2	52/55	94.6	0.41	0.522
Sheaths in xylem/sheaths in VB	10/102	9.8	Mar-55	5.5	0.41	0.522

^1^VB = vascular bundle.

## Discussion

Several studies have shown that *D. citri* nymphs are more efficient than adults in acquiring CLas from infected plants^9–11^, whereas the more mobile adults, especially those that acquired CLas during the nymphal stage, are more important for spreading this bacterium from tree to tree over medium and long distances^2,3,6,17,18^. Reasons for the greater ability of *D. citri* nymphs to acquire CLas from infected plants compared to the adults are unknown. The present work provides evidence that significant differences in the feeding behavior of nymphs and adults, especially with regard to phloem ingestion (E2), may explain this phenomenon. In 42-h long EPG recordings, nymphs were found to engage in phloem ingestion for 4 times as long as adults. Furthermore, the frequencies (no. of bouts) of the phloem penetration (D), phloem salivation (E1) and phloem ingestion (E2) were significantly higher for nymphs compared with adults. Our qPCR assays of psyllids following EPG recordings on infected plants indicated that 58% of nymphs and only 6% of adults acquired CLas during the 42 h acquisition period. These percentages are fairly close to the proportion of time spent on phloem ingestion by nymphs and adults (65 to 79, and 14 to 24%, respectively) during the test period. The mean CT values for CLas in infected psyllids were significantly lower in nymphs (30.6) compared to that of adults (36.5). These results strongly suggest that the greater frequency and duration of phloem ingestion by nymphs allow them to acquire a higher dose (titer) of CLas inoculum from infected plants compared with adults. We believe that both the duration and frequency of phloem ingestion bouts are important in this process, since several investigations indicated that CLas is unevenly distributed in citrus plant parts (stems or branches) and tissues^19–21^. Using immunoblot assays, Ding *et al*.^22^ reported that CLas was unevenly distributed in the phloem sieve tubes of citrus petioles. Also, most electron micrographs of CLas in phloem sieve elements of infected citrus plants contain much fewer bacterial cells compared with those from the phloem of infected dodder plants^16,23,24^. Thus, if CLas is unevenly distributed in each sieve tube as well as between various sieve elements, the ability of nymphs to tap more sieve elements (frequency of E2) and to spend a longer period in each element (mean duration of E2) may allow them to acquire more CLas inoculum from phloem compared with adults that spend a greater proportion of time engaged in xylem ingestion. It has been shown earlier that *D. citri* adults require more time than nymphs to acquire CLas from diseased plants, and that the acquisition access periods (AAP) is positively correlated with pathogen acquisition for adults^9,11^. This suggests that the frequency and duration of phloem ingestion activities, and the bacterial ‘load’ acquired by psyllids during feeding plays a role in determining the vector competency of the insect.

Yang *et al*.^25^ reported that both nymphs and adults of *D. citri* produced branched salivary sheaths but did not indicate any differences in regard to insect stage. Our histological study found that salivary sheaths produced by *D. citri* nymphs in citron leaves were more branched than those produced by adults. We also saw that the proportion of sheaths found at or close to the fibrous ring, believed to form a barrier to protect the vascular bundle^15,16^, was reduced for nymphs compared to those of adults, and that a higher percentage of nymphal stylet tracks reached the vascular bundle compared to those of adults (Table 4). It is possible that these three differences may be due to the smaller diameter of nymphal compared to adult stylets^16^. The thinner nymphal stylets may be more flexible, enabling them to branch in the mesophyll or other tissues and to navigate around the fibrous ring to reach the vascular bundle (Fig. 5E). This may also be the reason why nymphs penetrated more sieve elements (increased frequency of D-E1-E2 waveforms) by branching inside the phloem tissues (arrows in Fig. 5F). We hypothesize that increased frequency and duration of phloem penetration and ingestion by *D. citri* nymphs correlated with increased branching of the nymphal stylets, possibly related to higher flexibility of nymphal stylets, may explain the higher frequency of acquisition of CLas from infected citrus plants by nymphs compared with adults.

In contrast to *D. citri* and CLas, adults of the potato psyllid *Bactericera cockerelli* appear to be more efficient than 5^th^ instar nymphs in acquisition and transmission of *C*. Liberibacter solanacearum (CLso), the bacterium putatively associated with zebra chip disease in potatoes and “psyllid yellows disease in tomatoes^26^. Given this apparent contrast, it would be useful to conduct similar EPG studies on nymphs and adults of *B. cockerelli*.

The term “pathogen acquisition” has been used to indicate that the pathogen has been ingested from infected plant tissues into the vector’s gut (lumen). However, when the pathogen is transmitted in a persistent, circulative manner (as is CLas), acquisition usually refers to successful passage of the pathogen into the hemocoel since pathogens that remain in the gut lumen will not be transmitted^27^. When acquisition is defined in this wider sense, factors other than feeding behavior may be involved in the acquisition process. Once a large dose of CLas bacterium has been ingested by the psyllid, this bacterium has to enter and possibly multiply in the epithelial cells of the midgut, before invading and multiplying in other tissue^11,28^. It has been shown that CLas bacteria multiply faster and reach higher titer levels in *D. citri* upon acquisition by nymphs compared with CLas bacteria in adults^9,11^. Ramsey *et al*.^12^, based on a proteomic study of healthy and CLas-infected psyllids reported that differences in innate immunity may contribute to differences in acquisition/transmission efficiency of nymphs and adults. Other transmission barriers, e.g., entry or exit from/to the midgut or salivary glands in the psyllid vector, may also be at work in the CLas acquisition or transmission process^27,28^.

Relatively higher acquisition and transmission of pathogens by nymphs has been reported for several other insect-borne plant pathogens but feeding behavior of the vectors has not been elucidated in most of those cases^27,29^. Adult thrips (Thysanoptera) that vector tomato spotted wilt virus (TSWV, Bunyaviridae) can only transmit the virus following acquisition during the larval stages^30^. Kikkert *et al*.^31^ identified a 94-kDa protein in thrips that binds virus, but this protein was not present in the midgut of larval thrips and may be involved in virus specificity in other insect tissues.

In our work, the infection status of the host plant generally did not affect the frequency or duration of feeding waveforms except for the number of bouts of xylem ingestion (G) which was greater on infected compared with healthy plants (Table 1). The total duration of phloem and xylem ingestion bouts was influenced both by plant infection status and psyllid stage (Table 3). Total duration of phloem ingestion was greater on healthy plants. Conversely, total duration of xylem feeding was greater on infected plants. Additionally, adults engaged in more and longer bouts of xylem ingestion than nymphs. Adults also spent more time in non-probing (Np) or non-feeding activities compared with nymphs. This may be due to the fact that adults perform other behavioral activities that preclude feeding such as flight, mating and oviposition while nymphs engage in little or no movement once a satisfactory feeding site has been located. Consistent with our results, Cen *et al*.^32^ indicated that more *D. citri* adults probed xylem in CLas-infected citrus compared with healthy citrus. Also, psyllid adults did more xylem ingestion on resistant trifoliate citrus plants (*Poncirus trifoliata*) compared to sweet orange (*Citrus sinensis*)^33^. In our previous study, *D. citri* adults spent much more time in xylem ingestion on older citrus leaves compared with adults on young citrus flush^15^. The physiological condition of the plant, in addition to infection status, may influence the relative duration of xylem and phloem ingestion bouts by *D. citri* adults, and possibly by nymphs. The physiological condition of the insect may also be a factor. Bonani *et al*.^13^ and Spiller *et al*.^34^ suggested that psyllids and aphids use xylem feeding to obtain more water if starved prior to feeding.

This is the first electrical penetration graph (EPG) study comparing nymphs and adults of *D. citri* on healthy and infected citrus plants in relation to CLas acquisition from CLas-infected plants. We present evidence that supports the hypothesis that increased frequency and duration of phloem ingestion phase (E2 waveform) by *D. citri* nymphs may be a major factor in the greater ability of nymphs to acquire CLas, the putative causal agent of citrus greening/HLB, from infected citrus plants compared to adults. Our study provides another important step towards an understanding of the epidemiology of this most serious disease that is devastating citrus production worldwide. We hope that a better understanding of the feeding behavior of the psyllid and epidemiology of HLB will help towards better and innovative control strategies for both the psyllids and HLB disease.

## Methods

### Insects and plants used

#### Asian citrus psyllid

Adult *D. citri* were obtained from a colony established in 2000 at the USDA-ARS U.S. Horticultural Research Laboratory, Fort Pierce, FL. The psyllids were originally collected from citrus in the field and subsequently reared in a greenhouse in cages containing orange jasmine, *Murraya exotica* L. (*M. paniculata* auct. non.), and more recently *Citrus macrophylla* Wester as described by Skelley and Hoy^35^. The colony was confirmed quarterly to be free of CLas using qPCR^36^. All nymphs used in the EPG study were 4^th^ or 5^th^ instars; all adults used were 8 to 10-days-old. Previous work by us and others showed no differences in EPG waveforms produced by male or female *D. citri*^11,14^. Therefore, the sex of psyllids used in this study was not determined.

#### Plants

EPG studies were performed on *D. citri* nymphs or adults feeding on young fully expanded leaves of healthy or CLas-infected citron plants (*Citrus medica* L*.)* grown under greenhouse conditions. Plants were infected by grafting several months earlier, and infection was confirmed by qPCR^36^. Healthy and infected citron plants in 18.5 L pots were pruned to initiate flush. Infected plants with high pathogen titers (low CT values) were selected for the EPG recordings. Plants were washed and watered 24 h prior to the EPG experiment. Young leaves (soft, fully expanded, ca. 5 cm long and 3 cm wide) were selected for EPG recordings of *D. citri* and for subsequent qPCR and histological examination.

### Electrical Penetration Graph Recordings of *D. citri* nymphs and adults

EPG recordings were obtained using a DC-monitor, GIGA-8 model, EPG-Systems, Wageningen, the Netherlands^37^, adjusted to 50x gain. The analog signal was digitized through a DI-710 board and displayed using Windaq Lite ver. 2.40 software (Dataq Instruments Inc. Akron, OH, USA) on a Dell desktop computer. The EPG monitoring system was housed in a grounded Faraday cage in an environmentally controlled room under continuous lighted conditions. Temperature was set to 26 °C with 60–65% RH. Psyllid nymphs or adults were aspirated 4 h prior to the start of the experiment each day and were starved for this period inside glass vials. Psyllids were then placed in a freezer (−4 °C) for 45–60 s to immobilize them, then held by a plastic pipette tip connected to a gentle vacuum supply under a dissecting microscope. The psyllid was attached to a 25 µm-diam. gold wire (Sigmund Cohn Corp., Mt. Vernon, New York) by a droplet of silver conducting paint (Ladd Research Industries, Burlington, VT) applied to the pronotum. The gold wire lead was attached to a copper electrode (3 cm × 1 mm diameter) connected to the EPG probe. To complete the electrical circuit, a reference copper electrode (10 cm × 2 mm) was inserted into the soil medium near the base of the citrus plant. Psyllid nymphs and adults (Fig. S1) were restricted to the abaxial surface of the leaf, the preferred feeding site for nymphs^15^. Bonani *et al*.^13^ also used the abaxial side for his EPG study on *D. citri* adults. The feeding behaviors of individual *D. citri* adults and nymphs were monitored on infected citron plants (n = 33) or healthy plants (n = 18) for a continuous period of 42 h. This is much longer than EPG studies for *D. citri* reported previously. Our preliminary work indicated that *D. citri* nymphs are capable of continuous phloem ingestion for periods in excess of the 8 to 21 h of EPG recordings reported for adults^15^. Following the 42 h recordings, all tested insects were kept in individual vials with 90% ethanol, and leaves were stored in refrigerator until ready for extraction and qPCR.

Characterization of EPG waveforms was accomplished by visually identifying and annotating waveforms based on comparison to prior histological studies^13^. Windows Dataq waveform browser (Dataq Instruments Inc., Akron, OH) was used to annotate waveforms. The number and duration of waveform bouts were tabulated in an electronic spreadsheet. The waveforms were visually inspected for frequency patterns and annotated as non-probing (Np), mesophyll intercellular pathway (C), phloem penetration (D), phloem salivation (E1), phloem ingestion (E2) or xylem ingestion (G) phases. Statistical analysis was performed using JMP (v. 10, SAS Inc, Cary, NC). A 2 × 2 factorial design was used to evaluate main effects and interaction of psyllid age (nymph or adult) and plant infection status (healthy or infected) on the number of bouts corresponding to described waveforms and the total duration of each waveform during each 42 h recording.

### DNA extraction and qPCR of psyllids and citrus leaves

#### Asian citrus psyllid

Whole adult total nucleic acid was prepared using a Crude DNA isolation procedure previously described^11,28^. The DNA of individual insects was extracted using a Nucleospin Genomic DNA Kit (Macherey-Nagel, Bethlehem, PA). The insects (adults or nymphs) were ground individually in Macherey-Nagel Rack of Tube Strips (Cat. Number 740477.24, Macherey-Magel, Bethlehem, PA) by adding 3 (2.3 mm) chrome beads (Biospec Products, Bartlesville, OK) and homogenizing in a 2010 Geno/Grinder^®^ at 1600 RPMs for 4 minutes. DNA extraction was carried out according to the manufacturer’s instructions, and the DNA was finally collected in 30 µl of nuclease-free water. The samples were stored at −80 °C for further processing.

The following primer set^11^ amplifying a region of the Las 16 s rDNA (Genbank accession #DQ673424) was used to determine CLas titer: USHRL-CL1f:5′-CTTACCAGCCCTTGACATGTATAGGA-3′, and USHRL-CL1r:5′-TCCCTATAAAGTACCCAACATCTAGGTAAA-3′. Each 12.75 μl reaction consisted of the following reagents: 6.75 μL of GoTaq® Master Mix (Promega, Madison WI), 1 μl of Primer mix (F/R), and 5 μl crude psyllid DNA. Cycling parameters consisted of: hold at 95 °C for 2 min., 50 cycles (of 95 °C for 15 sec., 57 °C for 30 sec., 72 °C for 30 sec.), and an HRM melt from 74–88 °C, hold 1 sec. on 1^st^ step and 2 sec. on each subsequent step next. Two replicates from each from each insect were analyzed with qPCR in two independent runs, and the average CT (cycle threshold) value of two readings was used for each psyllid.

#### Citrus leaves

The leaves upon which psyllids had fed were immediately excised following EPG recordings. Midribs of inoculated leaves were processed for qPCR. Each midrib was separated from the leaf blade and chopped into very small pieces with a new sterile razor blade. Samples were placed in individual tubes and stored at −80 °C until further processing. Total plant CLas DNA was extracted using the Nucleo-Spin Plant II kit (Macherey-Nagel, Bethlehem, PA) with the exception that approximately 12 1.3-mm chrome steel beads (Biospec Products, Bartlesville, OK) were used. To these samples, 400 μl of lysis buffer and 10 μl of RNase was added prior to homogenization. Extraction was carried out according to the manufacturer’s protocols (Macherey-Nagel). After extraction, DNA was quantified by measuring absorbance at 260 nm using a Nanodrop 1000 (NanoDrop Products, Wilmington, DE), aliquoted, and diluted in nuclease-free water (QIAGEN, Valencia, CA). One hundred ng of DNA was used in each qPCR reaction. Samples were stored at −20 °C until used for PCR.

### Visualization of salivary sheaths produced by *D. citri* nymphs and adults

Adults and 3^rd^ or 4^th^ instar nymphs were caged separately in groups of >10 using muslin sleeves on 3 to 5 young leaves of healthy or CLas-infected citron plants for 4 days for adults (before any oviposited eggs could hatch) and 7 days for nymphs (before any nymphs could become adults). At the end of this feeding period, the leaves with the most nymphs or adults feeding on the lower (abaxial) side of the midrib were chosen for sectioning. The abaxial side was chosen so that it would be comparable to leaves used in the EPG study. The midrib was chosen because of the difficulty in cross-sectioning the smaller secondary veins. From each leaf, 2–3 small pieces of the midrib ca. 5 mm long, were cut with a sharp razor blade, fixed overnight in 4% paraformaldehyde in phosphate buffered saline (PBS), then washed 3 time in PBST (PBS + 0.1% Triton X 100). Each leaf section was placed in a drop of PBS on a microscope slide and sectioned by hand using a sharp razor blade to the thinnest possible sections under a stereomicroscope (at 20X or higher). These sections, determined by confocal microscopy to be ca. 50–70 μm thick^16^, were transferred gently (without staining) to a drop of Fluoro-Gel mounting medium (Electron Microscopy Sciences, Hatfield, PA, USA) on another microscope slide. Autofluorescence of salivary sheaths and surrounding leaf tissues^16^ was examined under UV light using an epifluorescence inverted microscope (Olympus IX70, with 4X or 10X objectives) fitted with a camera and an imaging program (CellSens software, Olympus, Tokyo, Japan). The occurrence, branching and position where the sheaths terminated (=termini) were recorded in each case. Approximately 100 to 150 sections from 2 to 3 different healthy or infected leaves fed on by ACP nymphs or adults were examined.

### Data availability

The datasets generated during and/or analyzed during the current study are available from the corresponding author upon request.

## Electronic supplementary material

Supplementary Figure S1
