# Supplementary figures and images for: Prolonged phloem ingestion by Diaphorina citri nymphs compared to adults is correlated with increased acquisition of citrus greening pathogen
Source: Sci Rep. 2018 Jul 9;8:10352. doi: 10.1038/s41598-018-28442-6 (PMC6037740; doi:10.1038/s41598-018-28442-6)

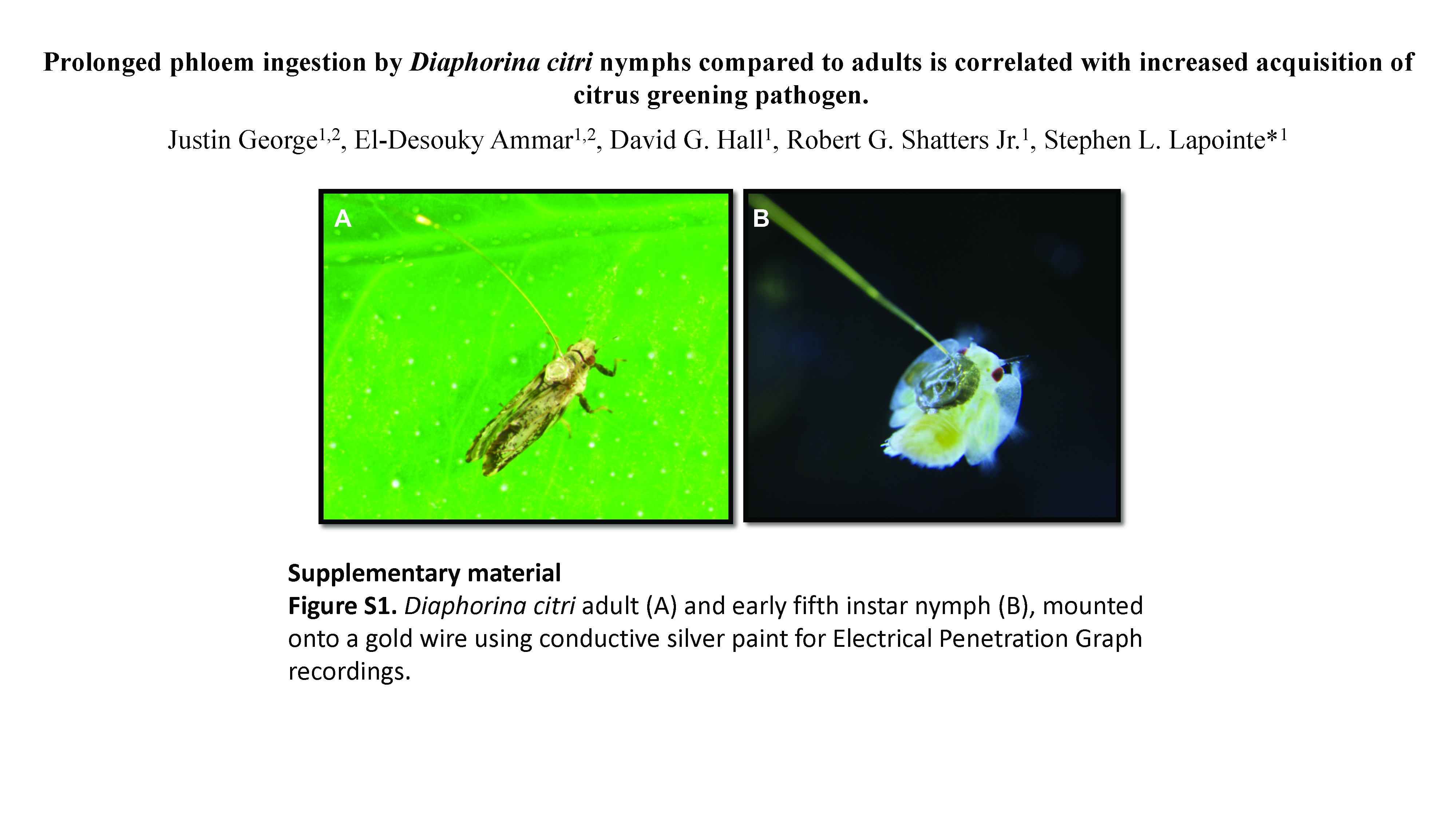

Supplement: Supplementary file 1 — Supplementary Figure S1 [file 41598_2018_28442_MOESM1_ESM.tif]
